# Delorme's Procedure for Complete Rectal Prolapse: A Study of Recurrence Patterns in the Long Term

**DOI:** 10.1155/2015/920154

**Published:** 2015-12-10

**Authors:** Carlos Placer, Jose M. Enriquez-Navascués, Ander Timoteo, Garazi Elorza, Nerea Borda, Lander Gallego, Yolanda Saralegui

**Affiliations:** Department of Colorectal Surgery, Division of General and Gastrointestinal Surgery, Donostia University Hospital, 20014 San Sebastián, Spain

## Abstract

*Introduction*. The objective of this study was to determine the recurrence rate and associated risk factors of full-thickness rectal prolapse in the long term after Delorme's procedure.* Patients and Methods*. The study involved adult patients with rectal prolapse treated with Delorme's surgery between 2000 and 2012 and followed up prospectively in an outpatient unit. We assessed epidemiological data, Wexner constipation and incontinence score, recurrence patterns, and risk factors. Data were analyzed by univariate and multivariate studies and follow-up was performed according to Kaplan-Meier technique. The primary outcome was recurrence.* Results*. A total of 42 patients, where 71.4% (*n* = 30) were women, with a median age of 76 years (IQR 66 to 86), underwent Delorme's surgery. The median follow-up was 85 months (IQR 28 to 132). There was no mortality, and morbidity was 9.5%. Recurrence occurred in five patients (12%) within 14 months after surgery. Actuarial recurrence at five years was 9.9%. According to the univariate analysis, constipation and concomitant pelvic floor repair were the only factors found to be associated with recurrence. Multivariate analysis showed no statistically significant differences among variables studied. Kaplan-Meier estimate revealed that constipation was associated with a higher risk of recurrence (log-rank test, *p* = 0.006).* Conclusions*. Delorme's procedure is a safe technique with an actuarial recurrence at five years of 9.9%. The outcomes obtained in this study support the performance of concomitant postanal repair and levatorplasty to reduce recurrences. Also, severe constipation is associated with a higher recurrence rate.

## 1. Introduction

Rectal prolapse is a condition with a substantial impact on patient's quality of life. The main clinical symptoms requiring treatment include fecal incontinence, constipation, rectal bleeding, mucous discharge, or the presence of the bulge itself. The goals of surgical treatment are to correct the prolapse and resolve or improve functional disorders (incontinence and constipation), with low morbi-mortality [1].

While a variety of abdominal and perineal procedures have been described to treat rectal prolapse, these are divided into abdominal and perineal approaches. It is considered that abdominal procedures carry a lower rate of recurrence and better functional outcomes but may entail an undesirable risk in young patients: fertility disorders in women and sexual function in men. In addition, the performance of abdominal surgery is technically more difficult in case of recurrence. Conversely, perineal approaches such as Delorme's or Altemeier;s procedure limit these risks at the expense of higher recurrence rates.

At present, laparoscopic surgery has become the treatment of choice for rectal prolapse in many Colorectal Units [2, 3]. However, perineal procedures are still performed in high-risk patients or in case of recurrence following abdominal surgery. A high BMI or the risk for nerve injury involved in abdominal surgery in young males may also lead to an indication of perineal surgery [4].

Although there is a range of ongoing randomized clinical trials (e.g., DeLoRes, Deliver, and Danish trial) whose results have not been published yet, at present there is not strong evidence of the superiority of a treatment over the others [5, 6].

The goal of this study was to assess recurrence rates in the long term following Delorme's procedure and identify the risk factors that might discourage this procedure.

## 2. Patients and Methods

This is an observational cohort study of patients undergoing Delorme's procedure for complete rectal prolapse at a tertiary hospital between January 2000 and December 2012. Patients' clinical records, physical examination data, and preoperative studies were prospectively collected. Imaging tests were occasionally performed (barium enema, transit time study, endorectal ultrasound, defecography, or pelvic magnetic resonance), as well as a colonoscopy and anorectal manometry. Patients were invited to take a picture of their prolapse when physical examination did not reveal the prolapse itself or there were doubts on the type of prolapse (complete or mucous) reported by the patient.

All patients received a preoperative enema and the administration of prophylactic antibiotic therapy with Metronidazole, Ciprofloxacin, or third-generation cephalosporins, as well as thromboembolic prophylaxis. Surgery was performed either under general or spinal hyperbaric anaesthesia in the lithotomy position. A urinary catheter was inserted. Surgery was performed as described in the literature [7, 8]. A dilution of adrenaline (1 : 200,000) was injected into the submucosal plane and a mucosectomy twice the length of the prolapse was performed. Reabsorbable suture was used for muscle plication and mucomucous anastomosis 1 cm above the anopectinate line. Park's posterior puborectal plicature or an anterior and posterior levatorplasty were selectively performed in patients with a patulous anus at the physical exploration, through the same incision by developing the intersphincteric plane and using nonreabsorbable monofilament suture. A fiber-supplemented diet was progressively introduced at 24 h following surgery. The urinary catheter was removed within the first day when the patient had no previous prostate disorders. Patients were discharged when they showed tolerance to oral diet, no/mild pain, and normal defecation.

Follow-up was performed in an outpatient clinic setting during the first two years following surgery; then, we contacted the patients or their GP during the first semester of 2013. Patient's baseline characteristics, postoperative complications, or recurrences were recorded. Incontinence and constipation were reassessed using a defecatory diary and Jorge and Wexner score [9].

Categorical variables were analyzed using either Chi-squared test or Fisher's test, as appropriate. Quantitative variables were analyzed by Mann-Whitney *U* test. Recurrences were assessed with Kaplan-Meier analysis. Risk factors for recurrence were identified by binary logistic regression and the Cox model. Statistical analysis was performed using SPSS 21.0 (SPSS Inc., Chicago, IL) software.

## 3. Results

A total of 30 women (71.4%) and 12 men (28.6%), with a median age of 76 years (IR 66 to 86), were included in the study. Two patients had undergone surgery for prolapse previously (a posterior rectopexy and a Frykman-Goldberg procedure). At baseline seven patients (16.6%) reported constipation and 15 had severe incontinence (35.7%). Fourteen women (46.6%) had undergone a hysterectomy and eight (26.6%) had some level of associated genital prolapse. As many as 29 patients (69.1%) had an ASA score III and 13 had an ASA score II (30.9%).

During surgery, hyperbaric spinal anaesthesia was used in 31 patients (75.8%) whereas general anaesthesia was used in 11 (26.2%). A levatorplasty was performed in seven patients (16.6%) (four postanal repairs and three anterior and posterior levatorplasty procedures). There was no mortality. Four patients (9.5%) experienced complications: two partial anastomotic dehiscences, a case of urinary retention, and a perineal haematoma. A patient (2.3%) required reintervention (resuture) for partial suture dehiscence. The average hospital stay was five days (IR 4 to 6). Sexual or urinary dysfunctions were not observed in any patient.

The median follow-up was 85 months (IR 28 to 132). Five recurrences (12%) were detected. All recurrences were diagnosed within the first 14 postoperative months: at 3 (one patient), 6 (one patient), 13 (two patients), 14 (one patient) months. Rerecurrence was not observed in any of the five patients who required reintervention after original Delorme's (3 re-Delorme, 1 Altemeier, and 1 laparoscopic ventral rectopexy). Neither de novo incontinence nor de novo constipation was observed during the follow-up.

**Figure 1:**
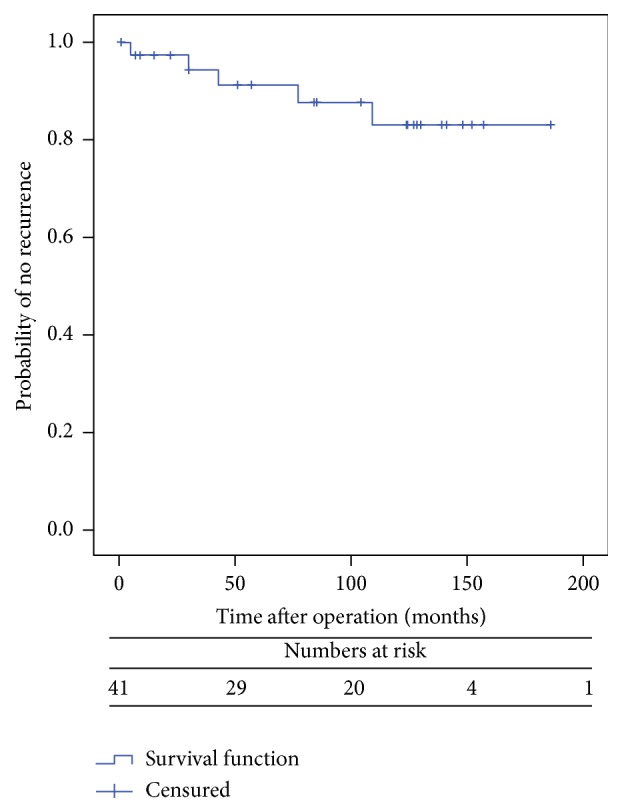
Probability of no recurrence after Delorme procedure (Global series).

**Figure 2:**
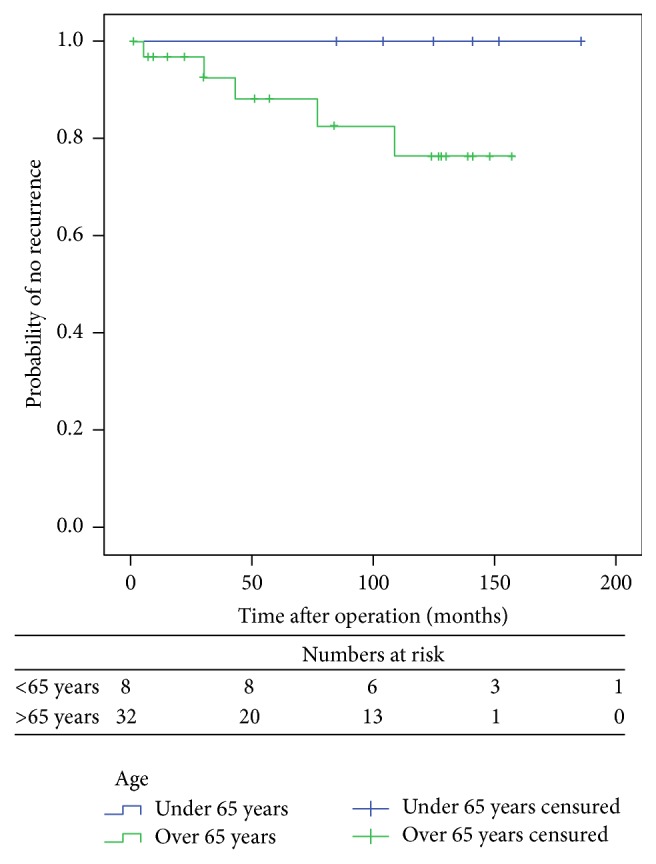
Comparison of probability of no recurrence with time for patients according to age (Kaplan-Meier method).

**Figure 3:**
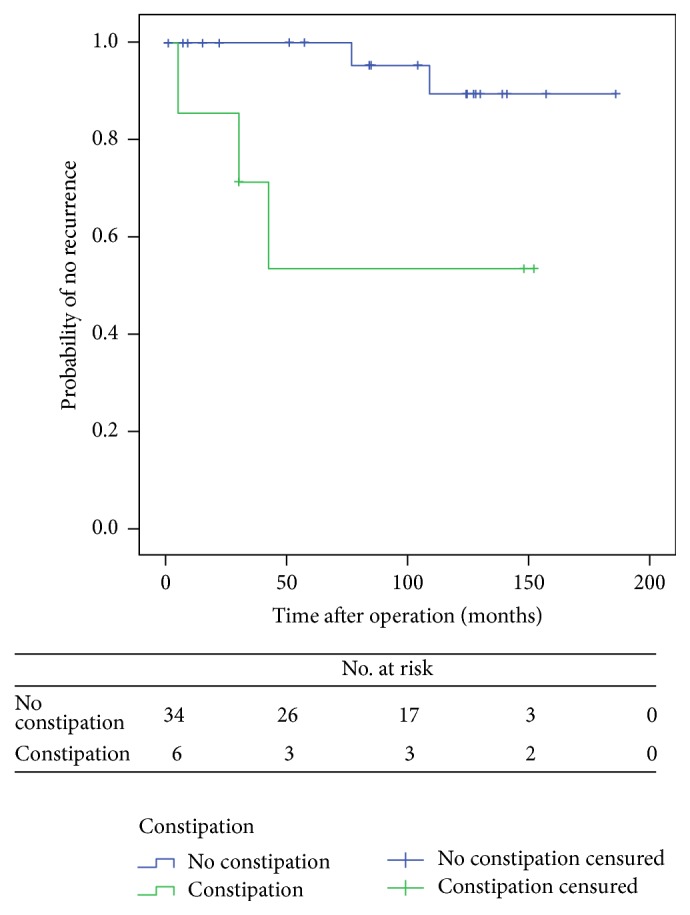
Comparison of probability of no recurrence with time for patients according to constipation (Kaplan-Meier method).

According to the univariate analysis, none of the main variables (i.e., age, sex, ASA score, previous hysterectomy, and postoperative complications) was found to be associated with recurrence (Table 1). However, significant differences were observed according to the type of anaesthesia (*p* = 0.013), constipation (*p* = 0.026), and performance of concomitant pelvic floor repair (*p* = 0.010). Recurrence was not observed in any patient aged <65 years (9/42), although differences were not statistically significant (*p* = 0.567). Also, of the five recurrences observed, four occurred in patients with a >5 cm prolapse, although differences were not statistically significant (*p* = 0.138). Multivariate analysis showed no statistically significant differences among variables. Kaplan-Meier estimate revealed that constipation was associated with a higher risk of recurrence (log-rank test, *p* = 0.006) (Figures 1, 2, and 3 and Table 2).

Outcomes for functional symptoms are shown in Table 3; as can be seen, during postoperative follow-up of patients, a tendency, to improvement in the degree of constipation, was observed, although it was not statistically significant. However a greater degree of constipation was observed in patients who had a later recurrence of the rectal prolapse. With respect to anal incontinence, no significant improvements were observed after the completion of the procedure Delorme.

## 4. Discussion

This study demonstrates that Delorme's operation is a safe procedure with very low mortality (0% in our series), a 9.5% morbidity, and an acceptable overall recurrence of 12% after a long median follow-up of seven years. Actuarial recurrence at five years was 9.9%. According to the univariate analysis, constipation and concomitant pelvic floor repair were the only factors found to be associated with recurrence, the former increasing the risk for recurrence and the latter reducing it. However, when multivariate analysis was performed these factors lost their individual influence.

The main limitation of this study is that it is a retrospective and observational study and some final controls were performed by the patient's general practitioner. On the other hand, the main strengths of this study are the long follow-up period, with a median follow-up above seven years, and the low levels of censored data during the study period. This is of special note since the recurrence rates reported in the literature are 47% lower as compared to those reported in an independent review [10].

Different factors have been reported to be associated with recurrence. Early recurrence seems to be clearly related to the execution of the technique and case selection. Two patients had recurrence within six months following surgery, may be due to defects of the technique or to underestimation of the prolapse. Partial resection of the prolapsed mucosa or a large prolapse requiring long muscle repair surgery may play a role in early recurrence.

Late recurrence is usually constant over the years. Variability of recurrence rates may be due to different size of the prolapses, associated pelvic disorders, follow-up periods, reinterventions, constipation, and case-mix [11, 12].

The two pathogenic factors associated with the development of complete rectal prolapse are recto-rectal invagination and a perineal herniation through a deep cul-de-sac of Douglas. It should be elucidated if the modification of these pathogenic factors through perineal surgery may have an influence on long-term outcomes.

Recurrence was not observed in any of the seven women who underwent concomitant posterior or total levatorplasty (7/30). Although differences were not statistically significant probably due to the small sample size, these results are consistent with those reported in the literature [8, 12, 13]. Youssef et al. conducted the only controlled randomized trial performed with 82 patients and found that complete anterior and posterior levatorplasty reduced recurrence from 14.28% to 2.43% [14]. Pelvic floor repair does not seem to influence invagination as a pathogenic mechanism. However, myorrhaphy and elevation of levator ani muscles may delay or prevent the formation of a new peritoneocele and hinder the descent of the longitudinal plication in Delorme's procedure. To avoid the protrusion of the apex of prolapses repaired by Delorme's procedure, Williams et al. designed the so-called express procedure by which rectal suspension is achieved using collagen strips [15]. The same conclusions can be drawn from other series treated with modified Altemeier's procedure combined with levatorplasty, although this technique can also avoid the “cul-de-sac of Douglas as a pathogenic factor” [16].

Many authors agree that the low recurrence rates among younger patients undergoing Delorme's procedure are due to the good state of their pelvic floor musculature as compared to elderly patients, who have a weak pelvic floor [17]. None of our patients aged <65 years had recurrence, but statistically significant differences were not observed due to the small sample size. As in our study, persistent constipation is considered a risk factor for recurrence in the literature. Although constipation improved in most patients as evidenced by a lower Wexner score, patients with persistent constipation are at a higher risk of early recurrence. Delorme's procedure improves constipation, as it reduces compliance and improves rectal sensation [18, 19].

Delorme's procedure can be performed for both primary and recurrent prolapse with good outcomes and low technical complexity [20]. No recurrences were observed in the long term in the three patients who underwent reintervention with Delorme's procedure due to recurrence.

Despite its methodological limitations, the recent multicenter controlled study Prosper has rekindled the debate about the effectiveness of the perineal approach [21]. However, this study shows that outcomes seem to depend more on the ability of the surgeon who performs the operation compared to the approach selected.

Although the preferred procedure for rectal prolapse in our Unit is laparoscopic ventral rectopexy, we consider perineal approach useful for particular cases of reintervention, high BMI, small prolapse, and absence of bowel dysfunction. In our opinion, age or surgical risk should not discourage an abdominal approach. A recent survey performed on the American College of Surgeons National Surgical Quality Improvement Program (NSQIP) revealed that the morbidity and mortality of laparoscopic surgery were similar to those of perineal surgery in elderly patients [22].

On the other hand, anterior and posterior repair of the pelvic floor should be systematically performed in all women requiring surgery for rectal prolapse in order to reduce recurrence rates in these patients. Patients with severe constipation are not ideal candidates for this technique unless abdominal surgery is not indicated for particular reasons. Young males undergoing surgery for rectal prolapse should be informed that abdominal surgery might cause pelvic nerve damage.

## Figures and Tables

**Table 1:** Baseline characteristics of rectal prolapse patients.

	Total (*n* = 42)	No recurrence (*n* = 37)	Recurrence (*n* = 5)	*p* value
Median age (years)	76	72	86	0.180^£^
Sex (men/women)	12/30	10/27	2/3	0.613^¥^
ASA (%)				
I	2 (4.8)	2 (5.5)	0 (0)	
II	12 (28.5)	11 (29.7)	1 (25)	
III	28 (66.7)	24 (64.8)	4 (75)	0.753^¥^
Hysterectomy^*∗*^ (yes/no)	14/16	11/16	3/0	0.090^¥^
Genital prolapse^*∗*^ (Y/N)	8/22	7/20	1/2	0.954^¥^
Incontinence (yes/no)	15/27	13/24	2/3	0.831^¥^
Constipation (yes/no)	7/35	4/33	3/2	0.026^¥^
Prolapse size				
<5 cms (%)	24 (57)	23 (61)	1 (20)	
>5 cms (%)	18 (43)	14 (39)	4 (80)	0.138^¥^
Anaesthesia (spinal/gral.)	31/11	30/7	1/4	0.013^¥^
Levatorplasty (yes/no)^*∗*^	7/23	7/20	0/3	0.010^¥^
Median hospital stay (days)	5	5	5	0.269^£^
Median follow-up (months)	85	104	43	0.144^£^
Complications (yes/no)	4/38	3/34	1/4	0.410^¥^

^*∗*^Data for 30 women; ^£^Mann-Whitney; ^*¥*^Chi^2^.

**Table 2:** Multivariate analysis.

	Total (*n* = 42)	No recurrence (*n* = 37)	Recurrence (*n* = 5)	*p* value	OR (IC 95%)
Anaesthesia (spinal/general)	31/11	30/7	1/4	0.047^¥^	0.07 (0.005–0.949)
Constipation (yes/no)	7/35	4/33	3/2	0.228^¥^	0.21 (0.017–2.656)
Prolapse size					
<5 cms (%)	24 (57)	23 (61)	1 (20)		
>5 cms (%)	18 (43)	14 (39)	4 (80)	0.483^¥^	0.414 (0.035–4.858)
Levatorplasty (yes/no)^*∗*^	7/23	7/20	0/3	1^¥^	0.464 (0–)
Hysterectomy^*∗*^ (yes/no)	14/16	11/16	3/0	1^¥^	0.730 (0–)

^*∗*^Data for 30 women; ^£^Mann-Whitney; ^*¥*^Chi^2^.

**Table 3:** Functional outcomes of Delorme's procedure.

	Total (*n* = 42)	No recurrence (*n* = 37)	Recurrence (*n* = 5)	*p* value
Constipation (Wexner)	7/35	4/33	3/2	0.026^¥^
Baseline (median, IR)	16.28 (8–21)	14.82 (6–19)	14.40 (8–19)	
Postoperative (median, IR)	12.82 (8–18)	13.82 (6–19)	11.33 (5–18)	
Incontinence (Wexner)	15/27	13/24	2/3	0.831^£^
Baseline (median, IR)	7.9 (4–12)	7.8 (4–12)	7.9 (4–12)	
Postoperative (median, IR)	7.4 (4–11)	7.6 (4–12)	7.4 (4–11)	

^*£*^Mann-Whitney; ^*¥*^Chi^2^.
